# Early intervention in post‐traumatic stress disorder without exposure to trauma memories using internet‐delivered cognitive therapy: A pilot case series

**DOI:** 10.1111/bjc.12419

**Published:** 2023-03-21

**Authors:** Graham R. Thew, Jennifer Wild, Anke Ehlers

**Affiliations:** ^1^ Department of Experimental Psychology University of Oxford Oxford UK; ^2^ Oxford Health NHS Foundation Trust Oxford UK

**Keywords:** case series, CBT, early intervention, IAPT, internet interventions, post‐traumatic stress disorder

## Abstract

**Objectives:**

Trauma‐focussed psychological interventions are the treatments of choice for post‐traumatic stress disorder (PTSD). As many clinical services receive high demand for PTSD interventions, strategies to improve treatment efficiency are needed. Some people seek help in the early phase post‐trauma, including as soon as the first few months. It is unclear whether all components of trauma‐focussed CBT are needed in this initial stage. Providing brief intervention in this early phase without work on trauma memories may be feasible and effective. This service evaluation study describes a case series of five participants experiencing PTSD following recent traumas.

**Methods:**

Participants completed a shortened 6‐week form of Internet‐delivered Cognitive Therapy for PTSD (iCT‐PTSD), which used fewer treatment modules and focussed primarily on psychoeducation about PTSD, and two key treatment components, ‘reclaiming your life’ and trigger discrimination. Unlike the full course of iCT‐PTSD, this format did not include working directly with trauma memories.

**Results:**

The intervention was associated with large reductions in symptoms of PTSD, depression and anxiety at the 6‐week timepoint, which were maintained at 3‐month follow‐up. Scores on the composite PTSD measure showed an average reduction of 91% between baseline and end of follow‐up. One client required an extension to the weekly phase of treatment and received further treatment modules. All were discharged after follow‐up and did not require further treatment.

**Conclusions:**

The findings provide preliminary evidence that this briefer format of iCT‐PTSD was of benefit for those seeking support following recent traumas. Further examination in a larger controlled study is required.

**Practitioner points**

- A briefer format of iCT‐PTSD, which focussed on decreasing unhelpful coping strategies, reclaiming life, and discrimination of memory triggers, and did not include exposure to trauma memories, was effective for the present participants.
- For clients with PTSD following recent traumas, brief interventions of this type may be beneficial, though larger controlled studies are required.

## INTRODUCTION

Post‐traumatic stress disorder (PTSD) is a globally prevalent mental health problem, which can arise following traumatic events, and is characterized by reexperiencing moments from the trauma, along with symptoms of avoidance, negative changes in thought or mood and marked changes in arousal and reactivity (American Psychiatric Association, 2013). Onset of such symptoms usually occurs soon after the trauma; A systematic review of studies of trauma‐exposed individuals found that on average, 25.4% of people met PTSD criteria at 1 month following the trauma (Santiago et al., 2013). This study indicated that trauma type (e.g., ‘intentional’ vs. ‘non‐intentional’) has a strong influence on the incidence of PTSD, though less so in the first 6‐months post‐trauma.

Trauma‐focussed cognitive behavioural treatments have strong evidence of efficacy in PTSD and are recommended as first‐line interventions (APA, 2017; ISTSS, 2019; NICE, 2018). These all involve exposure to the client's trauma memories and working with these memories to reduce reexperiencing. For example, in the *updating trauma memories* procedures in Cognitive Therapy for PTSD (CT‐PTSD: Ehlers & Clark, 2000; Ehlers et al., 2005), the therapist and patient identify the worst moments of the trauma and their meanings through narrative writing or imaginal reliving, identify information that contradicts these meanings or makes them less threatening in the present, and then update the trauma memory by linking the new information precisely to the relevant moment in memory.

Evidence suggests that in the early phase following trauma, many people show a natural improvement in PTSD symptoms (Lowe et al., 2021; Schell et al., 2004). Schell et al. (2004) showed that reexperiencing symptoms in particular show a reduction over the first 12 months. It might therefore be suggested that delaying treatment may be appropriate to allow this potential natural improvement to occur. However, evidence suggests that not all people show such natural improvement (Lowe et al., 2021) and that those with prominent hyperarousal symptoms have poorer outcomes at 12 months. Delaying treatment also risks the poorer outcomes associated with longer waits (Clark et al., 2018) or secondary problems such as substance misuse, and is unlikely to be acceptable to clients seeking support.

Studies of CBT interventions delivered in the first few months post‐trauma have generally shown positive outcomes, suggesting they are superior to no treatment, and promote recovery (McNally et al., 2003). However, it is less clear whether all the components of trauma‐focussed CBT are necessary for recent onset PTSD. Few studies have used Internet‐delivered interventions targeted to clients with PTSD in the early post‐trauma phase, with a systematic review finding just three studies of this type (Ennis et al., 2018). The authors concluded that while the results of these showed some evidence of efficacy in improving mental health outcomes, more studies, employing standardized measures of clinical outcome, are needed.

Cognitive Therapy for PTSD (CT‐PTSD: Ehlers et al., 2005) is a trauma‐focussed evidence‐based psychological therapy based on Ehlers and Clark's (2000) cognitive model of PTSD. It is one of the primary recommendations by the National Institute for Health and Care Excellence (NICE) for the treatment of adults with PTSD (NICE, 2018). It is routinely used in clinical services, including those that are part of the Improving Access to Psychological Therapies (IAPT) programme in England. An online, therapist‐guided version of this treatment (iCT‐PTSD) has shown promising results similar to those achieved with face‐to‐face CT‐PTSD (Ehlers et al., 2020, 2023; Wild et al., 2016). The online format may be an attractive treatment option in services such as IAPT, given the potential benefits for clients, such as the flexibility in when and where they work on their treatment, and the ability to work at their own pace or go back and read something again if they wish; and potential benefits for services, such as the reduced therapist time required per client, meaning more people can access treatment (Thew, 2020). This service evaluation study describes the treatment content and outcomes for five clients with PTSD related to recent traumas. They completed a shortened form of iCT‐PTSD focussing on psychoeducation about PTSD and unhelpful patterns of coping, and on two key treatment components, ‘Reclaiming your life’ and ‘Trigger discrimination’. Direct work on the trauma memory was not included in this treatment format. The aim was to evaluate whether this brief format of iCT‐PTSD could be helpful as an early intervention for those seeking help in the early phase post‐trauma.

## METHOD

### Participants

Participants were recruited from NHS IAPT services between October 19 and October 21. Over the recruitment period, clinicians alerted the study team to a total of nine referrals for consideration of eligibility. All had shown a recent onset of PTSD symptoms and considered this their main problem at the initial assessment in the service. A further assessment with the treating clinician was then completed to review eligibility. Clients were required to have experienced the onset of PTSD symptoms within the last 12 months and have the time, equipment and sufficient English‐language skills to undertake treatment using the iCT‐PTSD programme. Two clients did not meet PTSD criteria, but the remaining seven were eligible. All were offered and started treatment. Of these, five completed treatment and are described in the present study. One participant withdrew from treatment in Week 2 due to illness, and one requested to switch to face‐to‐face treatment in Week 3. Demographic and clinical details of each client, including an overview of their traumatic experience, are given in Table 1. For four clients, the index traumatic event had occurred within the past 12 months. The remaining client experienced a trauma 18 months prior, though symptom onset was delayed, starting 6 months ago. All participants provided informed consent for their treatment to be described and submitted for publication. Details have been changed to maintain anonymity.

**TABLE 1:** Participant demographic and clinical details.

Client	Gender	Age range	Time since trauma (months)	Clinical presentation
1	F	35–40	12	Client 1 witnessed her young daughter being resuscitated by paramedics and taken to hospital after developing a sudden illness at school. Her daughter survived but went on to be diagnosed with a chronic health problem. At the time of assessment, Client 1 was experiencing intrusive images of seeing her daughter lying on the floor looking grey and lifeless, with paramedics working all around her. She felt heartbroken and guilty, believing ‘I should have been there for her’. She felt anxious and tearful, overly alert and jumpy, with frequent worry that something bad would happen. This was particularly strong whenever her daughter was not with her. As a result, she always kept her phone to hand and checked it regularly for any missed calls from the school. She avoided leaving the house unless for work or going to or from the school. She described struggling to accept what had happened and her daughter's illness and felt that she was not able to move forward.
2	F	35–40	18	Client 2 stepped in to help an infant who had been injured in a public place and subsequently died. Symptoms of PTSD were not present immediately following the trauma, but showed a delayed onset following the birth of Client 2's own child 12 months later. At the time of assessment, she was experiencing intrusive images of the parents and infant, with many people around not knowing what to do. She felt agitated and irritable, had reduced concentration and felt that she was overreacting when stressed. She frequently worried that current situations were unsafe, questioning whether she should be taking her baby with her. She avoided going out alone with her baby or would check the area was safe first. She had been ruminating about the trauma and turning to food for comfort. As she had also stopped going to the gym, she was concerned this combination may lead to gaining weight.
3	F	25–30	4	Client 3 had a climbing accident, falling from a height onto concrete. She was immobile for several hours before paramedics were able to safely move her. At the time, she believed she had broken her back and neck and would never walk again. At assessment, she described frequent intrusive thoughts, particularly in the evenings, which were disrupting her sleep. She also experienced flashbacks of the falling sensation, which in some cases led to panic attacks. Her worries often centred around ‘how I could have prevented it’, and she would try to tell herself not to think about this. She avoided people and places that reminded her of what happened. Although she did not lose the ability to walk, the physical injuries she sustained meant it was no longer possible for her to do some activities she previously enjoyed such as hockey. She felt that overall she had become more reserved in her activities and was doing less exercise.
4	M	55–60	3	Client 4 was the victim of a mugging, during which he sustained a serious leg injury. At the time of assessment, he described struggling with nightmares and worrying about his safety, both of which were significantly affecting his sleep and subsequent energy levels. He felt guilty about what happened, with a strong fear of it happening again. He was experiencing emotional numbness much of the time and felt angry and restless in response to reminders of the event. He had been avoiding going near the place where it happened, and felt that he had stopped doing a number of activities he previously enjoyed, such as visiting family and spending time with friends. He was also unable to work at this time due to his injuries. He noted he had developed a checking routine to ensure doors and windows were fully closed and locked when leaving the house.
5	F	20–25	6	Client 5 was involved in a road traffic collision in which a car swerved onto her side of the road. At assessment, she described flashbacks and reexperiencing physical sensations such as the burning feeling on her arms where the airbag deployed. She experienced panic attacks linked to these flashbacks and nightmares that were leading her to delay sleep. She felt guilty about the accident, especially as her partner and young daughter had also been in the car, thinking ‘I should have gone a different route’. She had resumed driving, but was avoiding unnecessary trips or those involving unfamiliar locations or the location of the accident. This meant they were doing fewer enjoyable activities as a family. She was struggling with the reminders of the trauma generated by driving and described frequent scanning for danger and driving more cautiously.

### Procedure

iCT‐PTSD (Ehlers et al., 2020, 2023; see Wild et al., 2016) is a therapist‐guided Internet‐delivered intervention that replicates the content of Cognitive Therapy for PTSD (CT‐PTSD: Ehlers et al., 2005) using an online format. The main phase of treatment is typically delivered over 12 weeks and involves weekly brief (around 20 min) telephone calls with a designated therapist. This call is used to review the client's progress through the online treatment modules, guide the client through any difficulties and interventions that they may have found difficult to do on their own (e.g., finding updates for the worst moments in memory; behavioural experiments), and plan actions for the coming week. Support and guidance via written messaging and SMS is also provided between phone calls. The weekly phase of treatment is followed by up to 3 monthly booster calls to provide support with maintaining therapeutic gains. For the present study, a shorter 6‐week format was used, with some flexibility based on clinical need. The shorter format focussed primarily on psychoeducation about PTSD and patterns of coping that may impede recovery, plus two CT‐PTSD techniques that were thought to support the natural recovery process: (1) ‘Reclaiming your life’, which supports the client to identify and work towards resuming meaningful or rewarding activities and routines that have reduced or stopped following the trauma, and (2) trigger discrimination, which helps the client to recognize triggers in their everyday life, including subtle sensory triggers, and focus their attention on all the differences between the current trigger situation and the trauma (‘Then versus Now’; Ehlers et al. (2005)). Direct work on updating the worst moments of the trauma memory, which is a core component of the full course of iCT‐PTSD, was not included within this shorter treatment format. All other elements of the intervention, such as the level of therapist support and the booster phase, were the same as for standard iCT‐PTSD.

The treatment modules completed in the present study consisted of the following:

1. ‘Introducing the treatment’ provides an overview of PTSD and cognitive therapy and supports the client to reflect on their goals for therapy and any concerns they might have.
2. ‘It's all understandable’ provides psychoeducation on the impact of trauma and symptoms of PTSD, aiming to normalize its cognitive, emotional, physiological and behavioural consequences. It raises the idea that some of the behaviours that the client is likely to have used so far to deal with the trauma and memories may not be helpful and may actually impede change. A thought suppression experiment illustrates this point experientially.
3. ‘Reclaiming your life’, as described previously, is presented as a separate module, and the activities planned here are discussed and developed throughout the rest of treatment.
4. Trigger discrimination is introduced in a series of three brief modules, and the client is supported to practise applying the ‘Then versus Now’ technique on their specific triggers and record their progress using a separate page of the iCT‐PTSD website designed to facilitate this.
5. Towards the end of the weekly phase of treatment, all clients complete the ‘My Blueprint’ module. This guides them to reflect on and document their key observations and learning from therapy and to develop a plan for taking this forward and continuing to make progress towards their goals.
6. During the booster phase of treatment, a brief module called ‘Preparing for follow‐up’ is given prior to each monthly call. This encourages the client to consider and note their achievements and challenges experienced over the past month, which facilitates discussion of these and planning of appropriate next steps during the follow‐up call. Further details of iCT‐PTSD treatment content are provided in Wild et al. (2016), and video demonstrations of the key treatment components are available at https://oxcadatresources.com.

The present work took place across the COVID‐19 pandemic period. Some adaptations to the implementation of treatment techniques were therefore made where required, such as changes to the nature or location of reclaiming life activities or trigger discrimination practices (see Wild et al., 2020). All treatment was provided by the first author under supervision as part of routine service provision. The project was reviewed by the local NHS Trust Research and Development team and registered as a service evaluation project.

### Measures

Measures were completed weekly in line with standard IAPT procedures. These consisted of the measures that form the IAPT minimum dataset plus some additional process measures, described below. This set of measures is administered as standard within iCT‐PTSD and is completed within the programme, which provides graphs of scores over time to allow patients and therapists to monitor progress.

PTSD symptoms were measured using the PTSD Checklist for DSM‐5 (PCL‐5, Weathers et al., 2013) and the Impact of Events Scale—Revised (IES‐R, Weiss, 2007). In September 2020, IAPT services changed from using the IES‐R to the PCL‐5 as the principal PTSD symptom measure. This resulted in some missing data for these measures, though data completeness for providing at least one of these measures remained high (92%). We report results for each measure, along with a composite *z* score that averages the standardized scores for these two scales. Low mood was measured with the Patient Health Questionnaire 9 Item Version (PHQ‐9, Kroencke et al., 2001) and anxiety using the Generalised Anxiety Disorder Questionnaire (GAD‐7, Spitzer et al., 2006). Functional outcomes were assessed using the Work and Social Adjustment Scale (WSAS, Mundt et al., 2002). IAPT criteria were used to calculate reliable improvement (a drop of 10 or more on the PCL‐5, and/or a drop of 6 or more on the PHQ‐9, with neither measure showing an increase of equivalent magnitude), and recovery from PTSD (starting treatment above caseness on either the PCL‐5 [a score of 32 or more] or the PHQ‐9 [a score of 10 or more], and ending treatment below these thresholds on *both* measures).

The additional process measures assessed poor recall and disjointedness of trauma memories using the Trauma Memory Questionnaire—short version (MQ, Halligan et al., 2003), negative appraisals using the Post‐traumatic Cognitions Inventory—short version (PTCI, Foa et al., 1999) and safety behaviours using the short version of the Safety Behaviours Questionnaire (SBQ, Dunmore et al., 1999). Clients also completed the Responses to Intrusions Questionnaire—short version (RIQ, Clohessy & Ehlers, 1999; Murray et al., 2002), the Trait–State Dissociation Questionnaire—short version (TSDQ, Murray et al., 2002), and Insomnia Severity Index (ISI, Morin et al., 2011). Information and copies of process measures are available at https://oxcadatresources.com.

## RESULTS

Four clients completed treatment following the intended 6‐week structure, which incorporated some flexibility based on client availability, as well as a 3‐week treatment pause for one client due to a bereavement. Treatment for Client 5 was extended beyond the 6‐week format based on clinical need, and a further 5 weeks of treatment were provided. All participants completed the monthly booster phase and provided follow‐up questionnaire data at the end of this period. On average, participants had 7.40 phone calls with their therapist during the weekly phase of treatment (*SD* = 2.80), followed by 2.40 calls during the booster phase (*SD* = .50). The participants spent an average of 500 min (*SD* = 222.80) on the site (8.33 h) across the full treatment period. All participants completed the series of treatment modules described in the method. Client 5 completed some additional modules during the treatment extension. Treatment content for each participant is summarized in Table 2.

**TABLE 2:** Description of treatment for each client.

Client	Treatment description
1	*Reclaiming your life*: Activities for Client 1 included getting back to regular exercise classes and spending time further away from her ‘safety zone’. Through completing the treatment modules and through discussions with her therapist, she worked towards leaving the house every day, even if just short distances. She also worked to reduce her phone checking, experimenting with leaving her phone in her bag during most of the workday, as she did prior to the trauma. She also planned some days out with the family, which they had stopped doing since the trauma. *Triggers* included the lights and sirens of ambulances, the school room where the trauma took place, and her phone ringing. She was able to apply the Then vs Now technique and use this to practise working on these triggers, as well as applying it on a daily basis when faced with reminders in everyday life. This included times where she was in the school room, where she focussed on the key difference that ‘She is well and enjoys going to school, it is just where she goes to school now’. *Unhelpful coping behaviours*: Client 1 reported finding the ‘Train Station’ metaphor to be particularly helpful in supporting her to take an observer perspective on thoughts related to the trauma, like watching trains come and go from a station. *Client report of changes with treatment*: Client 1 described moving to a position of greater acceptance of her daughter's condition, recognizing that she now has much more support around this compared with the time of the trauma. She felt more accepting of the idea that it is ‘part of our life now’ and they can move forward.
2	*Reclaiming your life*: Activities for Client 2 included building towards regular exercise, such as going out for a brief run, or to the gym. Treatment also focussed on regaining some time for herself to do activities she used to enjoy such as watching films, painting and reading, as well as general self‐care and doing enjoyable activities with her family. *Triggers* included the sight and sound of helicopters and seeing locations that were similar to the trauma. Through discussion, we identified a further hidden trigger that of holding her baby in a particular position that was similar to the trauma memory. By working on these triggers, she described being more able to remind herself that the memory was in the past not the present. *Unhelpful coping behaviours*: Treatment also focussed on reducing other anxiety‐maintaining processes such as avoidance, scanning for danger when outside, and thought suppression. *Client report of changes with treatment*: Overall, Client 2 felt that treatment helped her to recognize that the trauma is now ‘just a memory of an event that happened’ and that there are more important areas of life she wants to focus on.
3	*Reclaiming your life*: Due to the physical injuries Client 3 sustained, reclaiming your life activities were adjusted based on her current level of mobility and rehabilitation. Swimming and going for walks were used for building up regular exercise, since hockey was not possible. Treatment also focussed on seeing friends more to reclaim the social element of hockey, as well as spending time on relaxing activities for herself. *Triggers* included ambulances, images or video of climbing, and being in a lying position in bed or on a sofa similar to her injured position after the accident. *Unhelpful coping behaviours*: Treatment also supported her with reducing thought suppression and establishing a regular sleep routine. *Client report of changes with treatment*: Client 3 reported finding the Then vs Now technique particularly helpful to practise working on these triggers and in managing them day‐to‐day. During the booster phase of treatment, she was no longer experiencing intrusive images of the event. Client 3 described that the case examples and videos in the treatment modules helped her to recognize that her experiences and symptoms were normal following a traumatic event.
4	*Reclaiming your life*: Activities for Client 4 included going out more regularly to visit friends and family, along with reinstating time for him to relax and do enjoyable activities. *Triggers* Most of his visits to friends involved passing through the area where the trauma occurred. Trigger discrimination was therefore used to support him when at the site of the trauma, which he did together with a friend initially, and subsequently became something he did regularly, including when alone. Other triggers included pain in his leg and the sight or sound of ambulances. *Unhelpful coping behaviours*: Treatment also included a behavioural experiment to explore reducing his checking routine before leaving the house and further discussions about the perpetrator to address rumination. *Client report of changes with treatment*: Client 4 described reaching a clearer recognition that the trauma ‘was random and not meant for me specifically’, which he found helpful. He reported improved sleep and by the end of treatment had been able to return to some of his work duties.
5	*Reclaiming your life*: Activities for Client 5 included taking trips out as a family, spending time with her daughter, going for short walks and scheduling time for herself to prioritize self‐care. All of these had reduced significantly since the trauma. *Triggers* included sudden noises, smells similar to that of the radiator and airbags during the trauma, plus a range of driving‐related reminders such as certain junctions, winding roads, cars braking suddenly and blue cars. Symptom improvement after the initial 6 weeks was limited, so treatment was extended in order to focus in more depth on the unhelpful coping behaviours outlined below. *Unhelpful coping behaviours*: Following discussion between client and therapist, it was agreed to focus principally on the driving‐related anxiety and safety behaviours and to work towards visiting the site of the trauma. Client 5 therefore completed two additional modules on ‘Understanding and Dealing with Risk’, which included psychoeducation on risk perception following trauma, estimation of objective risk and work on reducing safety behaviours such as scanning for danger. The ‘My Site Visit’ module was also completed, supporting Client 5 to view the site virtually using Google Street View, before planning an in‐person visit. *Client report of changes with treatment*: Client 5 reported that using Then vs Now at the site helped her recognize that ‘it's not happening anymore’ and provided her with a sense of closure. Direct work on the trauma memory was considered at this point but not clinically indicated by the questionnaire scores, so treatment moved into the follow‐up phase.

**FIGURE 1:**
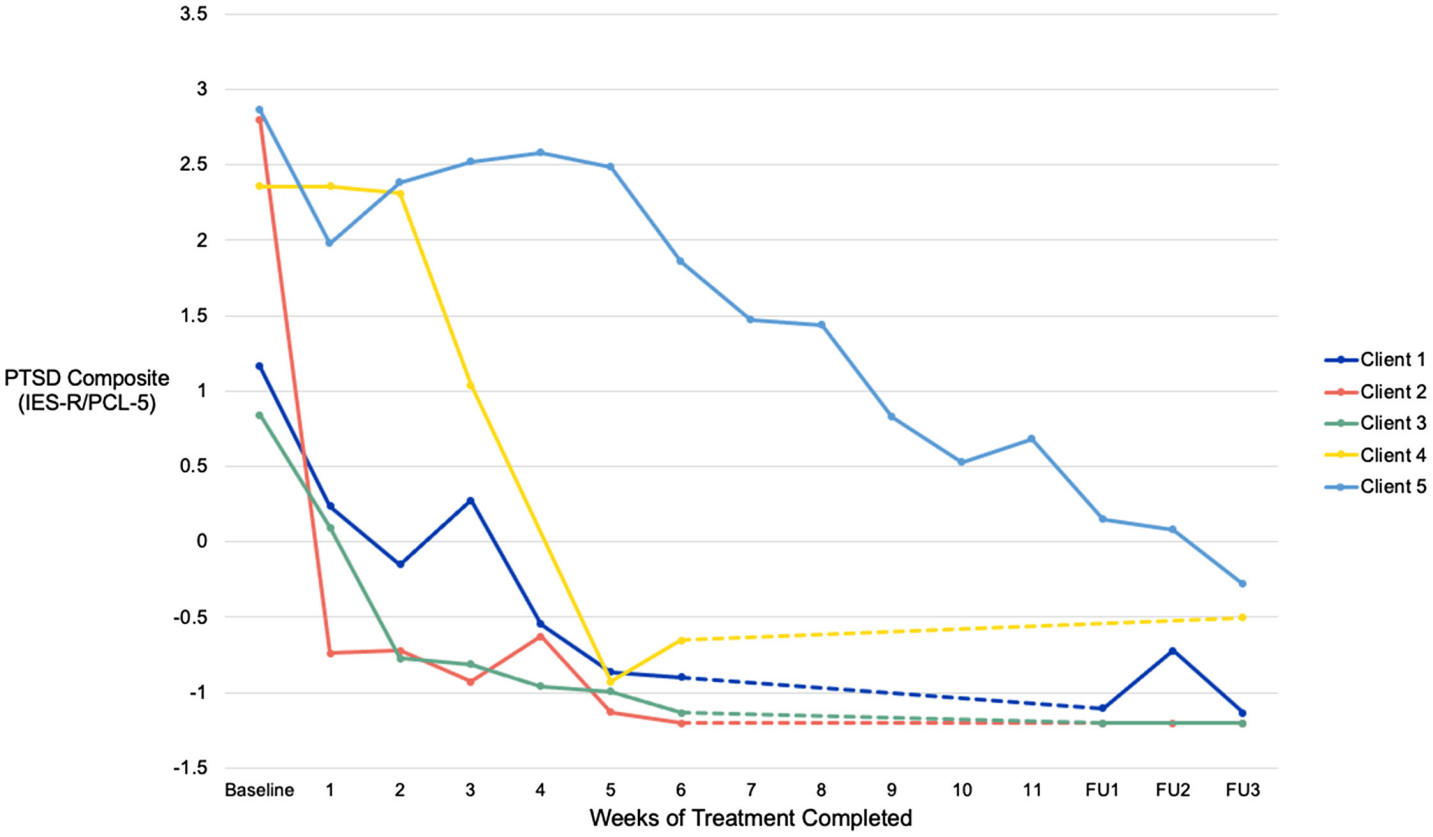
Scores on the PTSD symptom composite measure at each assessment. Composite formed as the average of the standardized scores on the IES‐R and PCL‐5. Week 6 represents the end of weekly treatment for all clients except Client 5 whose treatment was extended as shown.

Mean scores on symptom and process measures are shown in Table 3. Significance testing was not performed due to the small sample size, but estimates of effect size are indicated using Cohen's *d*. These indicate that the intervention was associated with large reductions in symptoms of PTSD, low mood and anxiety at the Week 6 timepoint and that these improvements were maintained at the 3‐month follow‐up. The results also indicated an improvement in functional outcomes on the WSAS, as well as reductions in the process variables of poor trauma memory recall and disjointedness, negative appraisals, safety behaviours, responses to intrusions, dissociation and sleep problems. Scores on the composite PTSD measure showed an average reduction of 91% between baseline and the end of the follow‐up period (see Figure 1).

**TABLE 3:** Scores on symptom and process measures.

Measure	Baseline, Mean (*SD*)	Week 6, Mean (*SD*)	End of follow‐up, Mean (*SD*)	Cohen's *d*
IES‐R	44.00 (15.56)	11.40 (18.49)	5.20 (6.69)	2.10
PCL‐5	49.67 (16.65)	11.80 (18.83)	4.60 (6.23)	2.27
PHQ‐9	13.00 (3.94)	3.00 (4.58)	1.60 (3.05)	2.54
GAD‐7	13.40 (3.51)	3.20 (6.06)	1.40 (3.13)	2.91
WSAS	12.00 (4.97)	6.60 (7.09)	2.60 (3.44)	1.09
MQ	9.20 (3.27)	3.00 (3.08)	1.80 (2.68)	1.90
RIQ	19.20 (7.19)	6.40 (8.02)	4.20 (5.02)	1.78
ISI	9.60 (9.24)	6.20 (7.60)	3.80 (4.49)	.37
SBQ	13.20 (4.32)	6.80 (6.22)	3.60 (2.61)	1.48
PTCI	60.20 (31.17)	40.00 (29.36)	36.80 (30.82)	.65
TSDQ	11.20 (13.44)	4.00 (7.87)	1.60 (2.61)	.54

*Note*: Cohen's d values represent change from baseline to Week 6.

Abbreviations: GAD‐7, Generalised Anxiety Disorder Questionnaire; IES‐R, Impact of Events Scale—Revised; ISI, Insomnia Severity Index; MQ, Trauma Memory Questionnaire; PCL‐5, PTSD Checklist for DSM‐5; PHQ‐9, Patient Health Questionnaire; PTCI, Post‐traumatic Cognitions Inventory; RIQ, Responses to Intrusions Questionnaire; SBQ, Safety Behaviours Questionnaire; TSDQ, Trait–State Dissociation Questionnaire; WSAS, Work and Social Adjustment Scale.

At Week 6, all participants showed reliable improvement based on IAPT criteria. In terms of IAPT recovery criteria, one participant started treatment just below the caseness thresholds on the PCL‐5 and PHQ‐9, so recovery could not be demonstrated. Of the remaining four participants, three met recovery criteria at Week 6, and all four at the end of follow‐up. No treatment‐related adverse events were reported. All participants were discharged following the iCT‐PTSD intervention as further treatment was not clinically indicated. At the time of writing, none had accessed further support from their IAPT service since this intervention.

## DISCUSSION

Overall, the findings provide preliminary evidence to suggest that this shortened early intervention format of iCT‐PTSD, which did not include any work on a trauma narrative or memory updating, was effective at reducing symptoms of PTSD, anxiety, depression and improving general functioning for clients with recent traumas. Changes on the process variables were also observed, which may suggest that changes in factors such as cognitions, safety behaviours, responses to intrusions and trauma memory qualities are involved in the process of clinical improvement (see also Ehlers et al., 2023). Besides psychoeducation about PTSD and raising awareness of unhelpful ways of coping and working towards changing them, the two principal components of this intervention were reclaiming your life and trigger discrimination. For reclaiming your life, the findings suggest it may have allowed participants to stop and review how things may have changed since the trauma and crucially to regain a sense of control over their activities and begin to reestablish a normal routine. It is possible that providing the reclaiming your life component in this early phase post‐trauma may be particularly helpful because it could prevent the longer‐term negative effects of losing meaningful and enjoyable activities and support people to review and make changes to their routines before patterns of avoidance become habitual. For trigger discrimination, it appeared that the early implementation of this technique was highly valued by participants, in that it gave them a tool to cope in the face of trauma reminders. The emotional impact of these reminders was generally one of the most acutely distressing symptoms they experienced. It seemed that learning about and practising the trigger discrimination technique may have helped participants develop confidence that they could cope with unexpected reminders. This in turn may have helped them feel more able to do activities they had been avoiding. The present results also indicate that trigger discrimination may have improved symptoms of hyperarousal, allowing them to reduce related safety behaviours such as scanning for danger. This finding is consistent with Schell et al. (2004) who found that the early targeting of hyperarousal symptoms was beneficial at supporting improvement across all other PTSD symptom clusters. For four of the five clients, these treatment components were sufficient to lead to recovery. Client 5 required more systematic work on driving with behavioural experiments including a site visit. Overall, these results support a modular approach for interventions in the first year following trauma where treatment modules from the iCT‐PTSD programme are added as needed.

Clinical services such as those in the IAPT programme in England receive many referrals for PTSD. Data from NHS Digital (2021) show that there were over 39,000 people seeking treatment for PTSD in 2020/21. Depending on capacity in individual services, an intervention such as this format of brief iCT‐PTSD may help to reduce lengthy waits for treatment. Longer wait times have been shown to be associated with poorer clinical outcomes (Clark et al., 2018). It is noted that some of the present clients showed significant early improvement after the first 1 or 2 weeks of treatment. This is consistent with previous studies (Wild et al., 2016) and suggests this approach potentially represents an efficient treatment option, though further controlled studies employing randomization and intention to treat analyses are needed.

In general, the findings are consistent with other studies implementing CBT interventions in the early phase post‐trauma, which have generally found that these are effective in comparison with no treatment (McNally et al., 2003). As a pilot study, it demonstrates in principle that a briefer iCT‐PTSD intervention with fewer treatment modules can be an effective early treatment option. However, the main limitation of the present study is the absence of control intervention, although the full iCT‐PTSD intervention has been compared with a wait list control condition with usual NHS care in other studies (Ehlers et al., 2020, 2023). At present, we cannot be sure whether for some of the clients in this case series, the observed symptom improvement may be due to a natural recovery process. However, it is unlikely that this applies to all five clients as the mean symptom severity at baseline was in the moderate to severe range and full recovery in the first year is more likely for those with less severe symptoms (e.g., Kleim et al., 2007). It is important to acknowledge that the present participants, and many others attending clinical services, sought help for their symptoms as they felt unable to manage, and that offering no intervention is unlikely to be acceptable to them. Other possible limitations include the fact that the present participants had mostly experienced single‐event traumas and previous trauma history was not assessed, so it is less clear whether the present findings would generalize to all trauma types seen within IAPT services. However, it is possible that early help‐seeking may be more common following a discrete index event. It is also possible that the brief approach could be beneficial for PTSD linked to more distant traumas, and further examination of this approach across different trauma characteristics would be helpful. PTSD was not confirmed using a structured diagnostic interview with the present participants and this should be considered for future studies. Lastly, it should be noted that most of the present work occurred in the context of the COVID‐19 pandemic and included periods of lockdown. As a result, some participants reported experiencing less frequent triggers in daily life as they were doing less. There were also some ways in which reclaiming your life activities were restricted, though alternatives were still planned (see Wild et al., 2020).

The present findings provide preliminary evidence to suggest that this early intervention format of iCT‐PTSD could have benefits for clients in terms of less need to wait and a shorter treatment that does not require working directly on the trauma memory. It might also have benefits for clinical services in that it could be potentially more efficient and may prevent the subsequent need for a more resource‐intensive full course of treatment, though this would need evaluating in a future study. Should future evaluation prove positive, it could be considered whether clients accessing support for more recent traumas may benefit from a separate treatment pathway in order to maximize the potential benefits of early intervention. A further empirical question would be which clinicians could offer this treatment. As trauma memory work is not included, it may be possible that the present intervention format could be delivered by staff such as Psychological Wellbeing Practitioners, who are not trained in PTSD treatment but have good experience in supporting remote interventions by telephone. This would require tailored training for staff delivering the intervention, with appropriate procedures in place to transition smoothly to a PTSD‐trained clinician should further input be clinically necessary.

In summary, the present case series highlights that intervening early post‐trauma can lead to highly positive clinical outcomes and that a briefer format of iCT‐PTSD might offer a promising route to achieve these. Further studies could examine the extent to which this intervention enhances a natural recovery process in the early phase post‐trauma and review the larger‐scale implementation of this treatment approach.

## AUTHOR CONTRIBUTIONS

**Anke Ehlers:** Conceptualization; supervision; writing – review and editing. **Graham R. Thew:** Conceptualization; data curation; formal analysis; investigation; methodology; project administration; visualization; writing – original draft; writing – review and editing. **Jennifer Wild:** Conceptualization; supervision; writing – review and editing.

## CONFLICT OF INTEREST STATEMENT

None to declare.

## Data Availability

The data that support the findings of this study are available on request from the corresponding author. The data are not publicly available due to privacy or ethical restrictions.
